# Vertical Cracks Excited in Lock-in Vibrothermography Experiments: Identification of Open and Inhomogeneous Heat Fluxes

**DOI:** 10.3390/s22062336

**Published:** 2022-03-17

**Authors:** Arantza Mendioroz, Alazne Castelo, Ricardo Celorrio, Agustín Salazar

**Affiliations:** 1 Departamento de Física Aplicada, Escuela de Ingeniería de Bilbao, Universidad del País Vasco UPV/EHU, Plaza Ingeniero Torres Quevedo 1, 48013 Bilbao, Spain; alazne.castelo@gmail.com (A.C.); agustin.salazar@ehu.es (A.S.); 2 Departamento de Matemática Aplicada, EINA/IUMA, Universidad de Zaragoza, Campus Río Ebro, Edificio Torres Quevedo, 50018 Zaragoza, Spain; celorrio@unizar.es

**Keywords:** crack characterization, lock-in vibrothermography, ultrasound-excited thermography, sonic-infrared, inverse problems, nondestructive testing

## Abstract

Lock-in vibrothermography has proven to be very useful to characterizing kissing cracks producing ideal, homogeneous, and compact heat sources. Here, we approach real situations by addressing the characterization of non-compact (strip-shaped) heat sources produced by open cracks and inhomogeneous fluxes. We propose combining lock-in vibrothermography data at several modulation frequencies in order to gather penetration and precision data. The approach consists in inverting surface temperature amplitude and phase data by means of a least-squares minimization algorithm without previous knowledge of the geometry of the heat source, only assuming knowledge of the vertical plane where it is confined. We propose a methodology to solve this ill-posed inverse problem by including in the objective function penalty terms based on the expected properties of the solution. These terms are described in a comprehensive and intuitive manner. Inversions of synthetic data show that the geometry of non-compact heat sources is identified correctly and that the contours are rounded due to the penalization. Inhomogeneous smoothly varying fluxes are also qualitatively retrieved, but steep variations of the flux are hard to recover. These findings are confirmed by inversions of experimental data taken on calibrated samples. The proposed methodology is capable of identifying heat sources generated in lock-in vibrothermography experiments.

## 1. Introduction

Thermographic non-destructive testing (NDT) methods have demonstrated a high potential for surface and subsurface defect detection and characterization [1]. Thermographic techniques consist in generating a thermal unbalance in the material and recording the evolution of the surface temperature distribution by means of an infrared camera. The thermal perturbation can be carried out by exciting the material with light (optically excited infrared thermography (IRT)), ultrasounds (vibrothermography, thermosonics, sonic IR), or electromagnetically (inductive thermography). The most popular modality of infrared thermography uses light to heat the material surface. The presence of defects perturbs the subsequent heat diffusion, giving rise to anomalies in the surface temperature distribution with respect to a sound material. Consequently, the signature of the defect needs to be identified in a pre-existent temperature field caused by the excitation. In this regard, vibrothermography has attracted a great deal of interest in recent times due to its defect-selective nature. In vibrothermography, the material is excited with high-amplitude ultrasounds. In non-viscoelastic materials, the bulk dissipation is small and the mechanical energy is converted into heat at cracks, mainly due to friction between the crack lips. This thermal energy diffuses in the material and eventually reaches the sample surface, producing a hot region above the defect, in a cold environment. For a comprehensive description of vibrothermography, see [2]. If compared to optically excited thermography, there is a double advantage in generating an internal heat source at the defect. First, the resulting surface temperature distribution is background-free and only due to the heat generated at the defect. Second, the heat travels only one way to reach the surface, which allows sensing deeper regions in the material. These advantages apply to any NDT method generating heat at defects, for instance, the identification of metallic inclusions embedded in an electrical insulator when the parts are excited by eddy currents (inductive thermography).

The heat generated at cracks in vibrothermography is generally non-uniform. Actually, in surface-breaking open cracks, the region where the crack lips are not in contact does not produce heat (unless an induced breathing mode brings the two surfaces into contact [3]) and, close to the crack border, the closure stresses might lock the crack asperities, thus preventing heat production [4]. Intermediate regions where the lips are in contact and in relative motion produce heat, generally with a non-uniform distribution. Accordingly, the geometry of this flux distribution is the information accessible from temperature data measured at the surface, rather than the crack geometry. 

The identification of the shape of internal heat sources from surface temperature data is a severely ill-posed inverse problem due to the diffusive nature of heat propagation. The strategies to solve this problem in a general form can be roughly categorized into least-squares minimization methods, statistical methods, and the new “virtual wave concept” method. In least-squares minimization, the cause of the observed temperature distribution (here, the inner heat source distribution) is identified by minimizing the squared L2-norm of the difference between the data and the prediction of the model (residual). The ill-posed character of the inverse problem makes this minimization unstable, and in order to find a sensible solution, the inversion needs to be regularized. An efficient strategy to stabilize the inversion and incorporate information on the characteristics of the solution consists in adding one or several terms to the residual that provide stability to the minimization. The minimization can be carried out by either global methods (neural networks [5], genetic algorithms [6], particle swarm optimization [7]), which search for the solution over large ranges of parameter values, or local methods (Gauss-type or conjugate gradient [8]), which modify the starting parameter values in a controlled way. Global methods are aimed at finding the rough global minimum but are less precise in finding the optimum solution and entail a high computational cost, whereas local methods may find the minimum more precisely but risk getting trapped at local minima.

In statistical methods [9,10,11], the solution is characterized by featuring the highest probability from a statistical point of view. Knowledge of the statistical uncertainty of the data set is required, as well as having a forward model in order to calculate the probability distribution to find the solution. Lastly, the recently developed virtual wave concept [12] is configured as a two-step problem. The first problem consists in calculating the so-called virtual wave, which can be understood as the wave equation solution counterpart of the true heat diffusion problem. Once found, in the second step, back-projection techniques allow finding the heat source distribution. 

The main difference between least-squares minimization and statistical methods versus the virtual wave concept is that the former need a physical model to describe the direct or forward process (calculation of the surface temperature from knowledge of the heat sources), whereas the later does not need modelization of the direct problem. 

So far, statistical methods and the virtual wave concept have been applied to characterize volumetric heat sources [12,13]. Least-squares minimization approaches have been implemented to characterize ideal, compact, and homogeneous vertical planar heat sources from lock-in vibrothermography data [14,15]. However, the heat generated by real cracks does not follow ideal, compact, and homogeneous distributions, unlike the sources treated in these previous works [14,15]. With the idea of approaching practical situations, in this work, we address the characterization of heat sources typically generated by real surface-breaking vertical cracks with half-penny shape, as well as inhomogeneous heat sources in vibrothermography experiments. We confine our study to the thermal diffusion problem, leaving aside the mechanisms that give rise to the heat generation. We focus on amplitude-modulated excitation and lock-in detection, as this modality is aimed at reducing the noise in the data, which is crucial in ill-posed inverse problems. In Section 2, we present the solution of the direct problem for the geometries addressed. In Section 3, we present a comprehensive overview of a regularized least-squares minimization approach, in order to give some insight on the meaning of regularization, and we describe the inversion algorithm. The potential and limitations of Lasso (L1) [16,17] and Total Variation (TV) [18,19] regularizations to identify open and inhomogeneous heat sources is shown in Section 4 by inverting synthetic data with added noise. In Section 5, we present the experimental set-up and inversions of experimental data, discussing the results. Finally, in Section 6, we summarize and conclude.

## 2. Direct Problem

**Figure 1:**
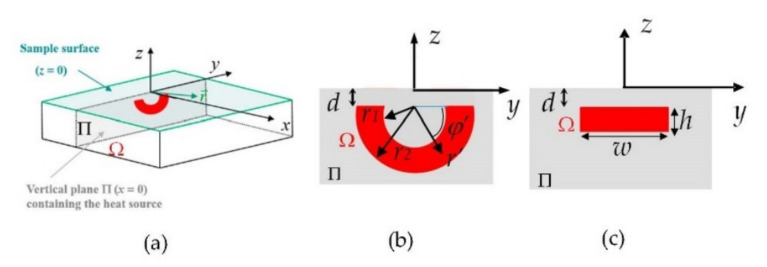
(**a**) Geometry of the problem, with heat sources in red; (**b**) detail of the geometry of the heat source, representing an open half-penny crack; (**c**) geometry of a rectangular heat source.

The direct problem consists in calculating the surface temperature distribution generated by a certain distribution of modulated heat sources (at frequency *f*, ω = 2π*f*) located in plane Π (*x* = 0) perpendicular to the sample surface (*z* = 0). We consider that the sample is semi-infinite in the *z* direction and infinite in *x* and *y* directions, with thermal conductivity *K* and diffusivity *D*. The geometry is depicted in Figure 1a.

Neglecting heat losses by convection and radiation, the complex temperature at the surface due to the thermal waves launched at frequency *f* from Ω can be calculated by integrating the contribution of point-like modulated heat sources in plane Π (confined in area Ω) [20]:

(1) $$T_{f}(x,y,0)=\iint_{\Pi }\frac{Q(\vec{r}’)}{4\pi K}\frac{e^{-q_{f}\left|\vec{r}-\vec{r}’\right|}}{\left|\vec{r}-\vec{r}’\right|}\;dS’=\iint_{\Omega }\frac{Q(\vec{r}’)}{4\pi K}\frac{e^{-q_{f}\left|\vec{r}-\vec{r}’\right|}}{\left|\vec{r}-\vec{r}’\right|}\;dS’$$

where $Q(\vec{r}’)$ is the position-dependent flux amplitude (null outside Ω) and $q_{f}=\sqrt{2\pi if/D}$ is the thermal wave vector. In order to describe the heat produced by half-penny surface-breaking cracks, we focus on heat sources featuring the shape of semi-circular bands of radii *r*_1_ and *r*_2_ (*r*_2_ > *r*_1_). For the sake of generality, we allow the heat source to be slightly buried, with the upper side located at a depth *d* with respect to the sample surface (Figure 1b). The complex surface temperature distribution for this case is written as follows:

(2) $$T_{f}\left(x,y,0\right)=\int_{r_{1}}^{r_{2}}\int_{0}^{\pi }\frac{Q(r’,\varphi ’)}{4\pi K}\frac{e^{-q_{f}\sqrt{x^{2}+{\left(y-r’\cos\varphi ’\right)}^{2}+{\left(d+r’\sin\varphi ’\right)}^{2}}}}{\sqrt{x^{2}+{\left(y-r’\cos\varphi ’\right)}^{2}+{\left(d+r’\sin\varphi ’\right)}^{2}}}r’dr’d\varphi ’$$

This expression also includes the case of kissing half-penny cracks, by making *r*_1_ = 0. 

For the sake of comparison, we also present inversions corresponding to other geometries. Just to give an example, we deal with rectangular heat sources of width *w* and height *h* buried at a depth *d* below the surface (Figure 1c). In this case, the expression of the surface temperature distribution is written as follows:

(3) $$T_{f}\left(x,y,0\right)=\int_{-w/2}^{w/2}\;\int_{-(d+h)}^{-d}\frac{Q(x’,y’)}{4\pi K}\frac{e^{-q_{f}\sqrt{x^{2}+{\left(y-y’\right)}^{2}+z{’}^{2}}}}{\sqrt{x^{2}+{\left(y-y’\right)}^{2}+z{’}^{2}}}dy’dz’$$

In Section 4, we present inversions of synthetic surface temperature data (amplitude and phase) calculated using Equations (2) and (3). For the inversion, we combine data obtained at modulation frequencies *f_k_* = 0.05, 0.1, 0.2, 0.4, 0.8, 1.6, 3.2, 6.4, and 12.8 Hz, corresponding to thermal diffusion lengths $\mu_{f}=\sqrt{D/\pi f}$ ranging from 0.3 to 5 mm: high frequencies provide sharp details, whereas low frequencies penetrate deeper in the material.

## 3. Inverse Problem

The general solution of the inverse problem consists in finding the heat flux distribution $Q(\vec{r}’)$ in plane Π, responsible for the observed (noisy) surface temperature data $T_{f_{k}}^{\delta }$, (*k* = 1,…, *k_max_*), *δ* being the noise level in the data (L2-norm of the noise).

This approach entails that:

1. The heat sources are known to be confined in a plane perpendicular to the surface (prior knowledge).
2. No specific geometry of the heat source is supposed.
3. The thermal properties of the material are known.
4. The shape of the spatial distribution of heat sources is unaffected by the modulation frequency.

Accordingly, even if the heat sources are known to be uniform within region Ω, the inversion is not a mere parameter estimation problem (*Q*, *r*_1_, *r*_2_, and *d* in Equation (1); *Q*, *w*, *h*, and *d* in Equation (2)) but entails meshing plane Π and determining the value of *Q* at each mesh node. This gives generality to the solution and is of practical interest, because the shape of the heat source is not known beforehand, but increases the difficulty of solving the problem. 

In this context, the formulation of the inverse problem in a least-squares sense consists in finding the *Q* distribution in plane Π that minimizes the L2-norm of the difference between the data and the calculated temperatures at each frequency, summed for all the modulation frequencies *f_k_*, *k* = 1, …, *k*_max_:

(4) $$R^{2}=\sum_{k=1}^{k_{\max}}{\left\|T_{f_{k}}(Q)-T_{f_{k}}^{\delta }\right\|}^{2}=\sum_{k=1}^{k_{\max}}{\left\|A_{f_{k}}Q_{f_{k}}-T_{f_{k}}^{\delta }\right\|}_{2}^{2}=\sum_{k=1}^{k_{\max}}{\left\|I_{f_{k}}^{}A_{f_{k}}Q-T_{f_{k}}^{\delta }\right\|}_{2}^{2}$$

Here, $A_{f_{k}}$ is the integral operator in Equation (1), and we have introduced a frequency-dependent heat source distribution $Q_{f_{k}}\left(r’\right)=I_{f_{k}}Q\left(r’\right)$ expressing $Q_{f_{k}}\left(r’\right)$ as the product of two factors: a normalized heat source distribution, $Q\left(r’\right)$, which, according to assumption 4, is common to all modulation frequencies, and a set of intensities, $I_{f_{k}}$, that only depend on the modulation frequency. This allows using different ultrasound amplitudes depending on the modulation frequency (typically, higher amplitude at high frequency, for which the signal is weaker). 

In this framework, in the inversion, the temperatures are not calculated using Equations (2) or (3) (or the corresponding expression for a particular geometry) but are obtained as the superposition of the point-like contributions of each mesh node in plane Π (Equation (1)). Accordingly, the number of unknowns in the inversion is significantly high (number of mesh nodes in plane Π). Given the ill-posed character of the inverse problem, the minimization of *R*^2^ is very unstable, and solving the problem requires stabilizing the inversion. A very popular method to stabilize ill-posed inverse problems is truncated singular-value decomposition (SVD). We opt for a different solution, which consists in minimizing a modified version of *R*^2^ by adding stabilizing terms to the right hand side of Equation (4), because this strategy allows introducing in the inversion prior information about the solution. In the next sub-section, following [8], we present a comprehensive and progressive introduction to the penalty terms that we incorporate in our inversion, taking truncated SVD as the starting point: from the well-known zero-order Tikhonov to more sophisticated functionals such as Lasso (L1-norm) or Total Variation (TV). We start with a quite general formulation, and later on, we particularize for the problem we are addressing. We have prioritized the smoothness of an intuitive description over rigor in formalism and notation. 

### 3.1. Regularization Functionals

#### 3.1.1. Truncated Singular-Value Decomposition 

We start by writing the direct problem in an operator form:

(5) AQ=T

where **A** is a linear matrix operator that maps the discretized heat source distribution **Q** in plane Π into the surface temperature data **T**. The least-squares problem is written as follows:

(6) $$A^{*}AQ=A^{*}T$$

where $A^{*}$ stands for a complex conjugate of A. The solution is:

(7) $$Q=(A^{*}A{)}^{-1}A^{*}T$$

If **A** has full column rank, (A^*^A)^−1^ exists, but if it was rank-deficient, (A^*^A)^−1^ would not exist and **Q** could not be calculated using Equation (7). The SVD method allows solving Equation (6) for rank-deficient matrices. Just as a reminder, in SVD, matrix **A** (*m* by *n*) is factored into 3 matrices:

(8) $$A=USV^{*}$$

where **U** is an *m* by *m* matrix whose columns are orthogonal vectors spanning the data space, **V** is an *n* by *n* matrix whose columns are orthogonal vectors spanning the model space, and **S** is an *m* by *n* diagonal matrix whose diagonal elements *s_i_* (singular values) are arranged in decreasing order. If only the first *p* singular values are non-zero (*p* < *m*), **S** can written as

(9) $$S=\left[\begin{array}{cc} S_{p} & 0\\ 0 & 0 \end{array}\right]$$

and Equation (8) can be simplified to $A=U_{p}\;S_{p}\;V_{p}{}^{*}$, where **U_p_** and **V_p_** denote the matrices whose columns are the first *p* columns of **U** and **V**, respectively. The SVD can be used to compute a generalized inverse of **A**, the so-called Moore–Penrose pseudoinverse, **A**^†^,

(10) $$A^{†}={\left(A^{*}A\right)}^{-1}A^{*}=V_{p}\;S_{p}^{-1}\;U_{p}^{*}$$

which always exists. The pseudoinverse solution is then:

(11) $$Q^{†}=A^{†}T=V_{p}\;S_{p}^{-1}\;U_{p}^{*}\;T$$

In an explicit form, the pseudo-inverse is written as follows:

(12) $$Q^{†}=\overset{p}{\underset{i=1}{\sum }}\frac{U_{.,i}^{*}\;T}{s_{i}}V_{.,i}$$

where $U_{.,i}$ and $V_{.,i}$ represent each of the *p* columns of **U_p_** and **V_p_**, respectively. 

Equation (12) presents the solution as a linear combination of model space vectors, multiplied by factors containing the corresponding singular value *s_i_* at the denominator. The summation may include terms with very small singular values that give rise to very large coefficients for the corresponding high-frequency model space vectors $V_{.,i}$, which may eventually dominate the solution, acting as noise amplifiers. 

A natural way to stabilize the solution consists in discarding Equation (12) model space vectors with very small associated singular values. This is so-called truncated SVD regularization. However, this stability comes at the expense of reducing the accuracy of the solution. Therefore, the criterion to discard model space vectors must be a trade-off between stability and accuracy of the solution.

#### 3.1.2. Zero-Order Tikhonov Regularization

A successful solution of an inverse problem generally involves reformulation as an approximate well-posed problem. The zero-order Tikhonov regularization [21] modifies the least-squares equation by adding a smoothing term in order to reduce the unstable effects of noise in the data. 

When the data are noisy, there might be many solutions that adequately fit the data, so that ||**AQ** − **T**||_2_ is small enough. In zero-order Tikhonov regularization, the solutions are sought among those that meet ||**AQ** − **T**||_2_ ≤ *δ* (*δ* being a specific residual misfit value), selecting the one that minimizes the L2-norm of **Q**:

(13) $$\min{\left\|Q\right\|}_{2},\;\mathrm{subject}\;\mathrm{to}\;{\left\|AQ-T\right\|}_{2}\leq \delta$$

Introducing zero-order Tikhonov regularization (for a specific regularization parameter *α_TK_*), the problem formulated in Equation (13) can be written as the minimization of:

(14) $$R^{2}={\left\|AQ-T\right\|}_{2}^{2}+\alpha_{TK}^{}{\left\|Q\right\|}_{2}^{2}$$

Equation (14) is the so-called objective function, and the first and second terms on the right hand side are the so-called discrepancy term and regularization term, respectively. The regularization term is the product of a regularization parameter, *α_TK_*, and a regularization functional, ${\left\|Q\right\|}_{2}^{2}$ in this case. The larger the *α_TK_*, the more powerful the regularization and the larger the error in the solution. We describe our strategy to determine the optimum value of the regularization parameter in Section 3.2.1. 

The zero-order Tikhonov solution is equivalent to an ordinary least-squares problem augmented according to:

(15) $$Q^{\alpha_{TK}}=\arg\underset{Q\in {\mathbb{R}}^{n}}{\min}{\left\|\left[\begin{array}{c} A\\ \sqrt{\alpha_{TK}}I \end{array}\right]Q-\left[\begin{array}{c} T\\ 0 \end{array}\right]\right\|}_{2}^{2}=\arg\underset{Q\in {\mathbb{R}}^{n}}{\min}{\left\|A_{aug}Q-\left[\begin{array}{c} T\\ 0 \end{array}\right]\right\|}_{2}^{2}$$

The size of **A** remains *m* by *n*, and **I** is the *n* by *n* identity matrix. As long as *α_TK_* is non-zero, the last *n* rows of matrix **A***_aug_* are linearly independent, so Equation (15) represents a full-rank least-squares problem that can be solved by its normal equations:

(16) $$A_{aug}^{*}A_{aug}Q^{\alpha_{TK}}=A_{aug}^{*}T$$

Using the SVD of **A** and following the steps indicated in Section 3.1.1, the solution can be written as:

(17) $$Q^{\alpha_{TK}}=\overset{k}{\underset{i=1}{\sum }}\frac{s_{i}}{s_{i}{}^{2}+\alpha_{TK}^{}}U_{\cdot ,i}^{*}\;T\;V_{\cdot ,i}$$

where *k* = min (*m*,*n*), and all non-zero singular values and vectors are included. Equation (17) can be rewritten as:

(18) $$Q^{\alpha_{TK}}=\overset{k}{\underset{i=1}{\sum }}\frac{s_{i}{}^{2}}{s_{i}{}^{2}+\alpha_{TK}^{}}\frac{U_{.,i}^{*}\;T}{s_{i}}V_{.,i}=\overset{k}{\underset{i=1}{\sum }}f_{i}\frac{U_{.,i}^{*}\;T}{s_{i}}V_{.,i}$$

where $f_{i}=s_{i}^{2}/(s_{i}^{2}+\alpha_{TK})$ are the so-called filter factors, which control the contribution of the different terms to the sum, in the fashion of a low-pass filter. Comparison of Equations (12) and (18) shows that the penalization of different model space vectors depends on the relation between *α_TK_* and their associated singular values. Accordingly, the degree of regularization varies between two limiting cases: for *s_i_* >> *α_TK_*, *f_i_* ≈ 1, and the contribution of the corresponding model space vectors in Equation (18) remains the same as in Equation (12), whereas for *s_i_* << *α_TK_*, *f_i_* ≈ 0, i.e., the associated model space vectors are highly damped. For intermediate singular values, as *s_i_* decreases, *f_i_* produces a decreasing contribution of the corresponding model space vectors. The result is a filtering of model space vectors with small singular values softer than applying truncated SVD. As a consequence, zero-order Tikhonov regularization produces a smooth solution, since sharp, high-frequency model space vectors are filtered out. 

Finally, let us mention that it is also possible to apply penalty terms that minimize the L2-norm of the first or second derivatives of the solution, rather than the L2-norm of solution itself. These are the so-called first- and second-order Tikhonov functionals, which are mentioned in the next section.

#### 3.1.3. Lasso and Total Variation Regularizations

Focusing now on the particular inverse problem that we are addressing, we come back to Equation (4). As mentioned at the beginning of this section, our goal is to retrieve the vertical heat source distribution *Q* that minimizes a regularized version of the squared L2-norm in Equation (4). In practice, this is carried out by meshing plane Π with *n* nodes. 

If a zero-order Tikhonov penalty term is applied, the regularized version of Equation (4) is written as follows:

(19) $$R_{}^{2}=\sum_{k=1}^{k_{\max}}{\left\|I_{f_{k}}^{}A_{f_{k}}Q^{}-T_{f_{k}}^{\delta }\right\|}_{2}^{2}+\alpha_{TK}TK\left(Q\right)\;\mathrm{with}\;TK\left(Q\right)=\iint_{\Pi }{\left|Q\right|}^{2}dS\approx \sum_{i=1}^{n}{\left|Q_{i}\right|}^{2}\Delta S$$

Zero-order Tikhonov regularization penalizes all nodes in plane Π equally, as it applies the same regularization parameter to each one, with no further information regarding possible locations of the heat sources. However, in order to optimize the degree of regularization, other non-linear regularization procedures based on local information can be implemented, aimed at performing a position-dependent penalization.

Lasso (L1) [16,17] and total variation [18,19] regularization methods allow performing a position-dependent penalization by assigning a different regularization parameter to each node in plane Π, which, in turn, is made feasible by implementing iterative methods that make use of the heat source distribution retrieved in a previous iteration. This way, it is possible to have an idea of which nodes need to be penalized more in a following iteration, in order to force some of them to remain damped and keep others dominating the solution. 

Let us consider a penalty term based on a zero-order Tikhonov functional, as the one considered in Equation (19), but with a regularization parameter that takes into account the solution in a previous iteration:

(20) $$\alpha_{TK_{i}}=\alpha_{L1}\frac{1}{\left|Q_{i,k-1}^{\alpha_{L1}}\right|}$$

where *i* denotes the node in plane Π, *k* is the iteration, and we assume that $\left|Q_{i,k-1}^{\alpha_{L1}}\right|\neq 0$. The explicit expansion of this new discretized penalty term for all nodes is written as follows:

(21) $$\begin{array}{l} \sum_{i=1}^{n}\alpha_{TK_{i}}{\left|Q_{i,k}^{\alpha_{L1}}\right|}^{2}\Delta S=\sum_{i=1}^{n}\alpha_{L1}\frac{{\left|Q_{i,k}^{\alpha_{L1}}\right|}^{2}}{\left|Q_{i,k-1}^{\alpha_{L1}}\right|}\Delta S=\\ \;\;\;\;\;\;\;\;\;\;\;\;\;\;\;\;\;\;\;\;\alpha_{L1}\left(\frac{1}{\left|Q_{1,k-1}^{\alpha_{L1}}\right|}{\left|Q_{1,k}^{\alpha_{L1}}\right|}^{2}+\frac{1}{\left|Q_{2,k-1}^{\alpha_{L1}}\right|}{\left|Q_{2,k}^{\alpha_{L1}}\right|}^{2}+\ldots +\frac{1}{\left|Q_{n,k-1}^{\alpha_{L1}}\right|}{\left|Q_{n,k}^{\alpha_{L1}}\right|}^{2}\right)\Delta S. \end{array}$$

As can be seen, despite $\alpha_{L1}$ being common for all terms, each term is affected by a different penalization, because the $Q^{\alpha_{L1}}$ values are divided by the local values obtained in the previous iteration. In this way, if $\left|Q_{i,k-1}^{\alpha_{L1}}\right|$ is small and thus $1/\left|Q_{i,k-1}^{\alpha_{L1}}\right|$ is large, then $\left|Q_{i,k}^{\alpha_{L1}}\right|$ is forced to remain small. Otherwise, $1/\left|Q_{i,k-1}^{\alpha_{L1}}\right|$ is small and $\left|Q_{i,k}^{\delta ,\alpha_{L1}}\right|$ is free to increase or vary.

Over iterations, eventually $\left|Q_{i,k-1}^{\alpha_{L1}}\right|\approx \left|Q_{i,k}^{\alpha_{L1}}\right|$, and the penalty term in Equation (21) approaches:

(22) $$\sum_{i=1}^{n}\alpha_{TK_{i,j}}{\left|Q_{i,k}^{\alpha_{L1}}\right|}^{2}\Delta S\approx \alpha_{L1}\left(\left|Q_{1,k}^{\alpha_{L1}}\right|+\left|Q_{2,k}^{\alpha_{L1}}\right|+\ldots +\left|Q_{n,k}^{\alpha_{L1}}\right|\right)\Delta S$$

which represents the L1-norm of $Q^{\alpha_{L1}}$ multiplied by the regularization parameter $\alpha_{L1}$. Thus, penalizing the least-squares minimization with a penalty term based on the lasso (*L*1) functional:

(23) $$L1\left(Q\right)=\iint_{\Pi }\left|Q\right|\;dS=\left\|Q\right\|{\;}_{1}≃\underset{k\to \infty }{\lim}\iint_{\Pi }\frac{{\left|Q_{k}\right|}^{2}}{\sqrt{\varepsilon +{\left|Q_{k-1}\right|}^{2}}}\;dS$$

can be interpreted as performing a position-dependent penalization of zero-order Tikhonov penalization. The presence of a small constant ε in the denominator of Equation (23) is aimed at avoiding computing errors when $\left|Q_{k-1}\right|\approx 0$. Equations (21) and (22) describe the lagged fix-point iterations algorithm that can be used to approximate the non-quadratic *L*1 penalty term defined in Equation (23). 

Regularization with a total variation penalty term:

(24) $$TV\left(Q\right)=\iint_{\Pi }\left|\nabla Q\right|\;dS={\left\|\nabla Q\right\|}_{\;1}$$

is based on the same principle as *L*1, but acting over $\left|\nabla Q\right|$ instead of $\left|Q\right|$. It can be interpreted as the implementation of a first-order Tikhonov functional with a position-dependent regularization parameter. The lasso functional penalizes the L1 norm of the solution, and *TV* penalizes the *L*1 norm of the gradient of the solution. In practice, the main difference between *L*1 and *TV* for the solution of the inverse problem is that *L*1 favours sparse solutions in plane Π (compressive sensing effect), whereas *TV* favours solutions with areas of null derivatives, which yields blocky solutions. The combination of both is appropriate to characterize the confined heat sources representing cracks that we are seeking. Similarly to Equation (22), which approximates the L1-norm of the solution, since *TV* is a non-quadratic operator, it can be approximated from first-order Tikhonov penalty functional using lagged fix-point iterations:

(25) $$TV\left(Q\right)≃\underset{k\to \infty }{\lim}\iint_{\Pi }\frac{{\left|\nabla Q_{k}\right|}^{2}}{\sqrt{\varepsilon +{\left|\nabla Q_{k-1}\right|}^{2}}}\;dS=\underset{k\to \infty }{\lim}\iint_{\Pi }\frac{{\left(\partial_{y}Q_{k}\right)}^{2}+{\left(\partial_{z}Q_{k}\right)}^{2}}{\sqrt{\varepsilon +{\left(\partial_{y}Q_{k-1}\right)}^{2}+{\left(\partial_{z}Q_{k-1}\right)}^{2}}}\;dS$$

Throughout this section, we have seen that particular regularization functionals produce specific types of solutions: zero-order Tikhonov yields smooth solutions, *TV* generates blocky functions, and lasso produces a compressive sensing effect. This indicates that, in ill-posed inverse problems, given some prior knowledge of the properties of the solution, the mere selection of the penalty functional is a tool to incorporate this prior information in the inversion. 

According to the previous results, we stabilize our inversion by penalizing the minimization with two functionals based on *TV* and *L*1, plus an auxiliary zero-order Tikhonov penalty term. The properties of *TV* and *L*1 motivate this selection, as we seek confined heat sources produced at cracks in well-defined areas. The regularized version of Equation (4) to be minimized is written as follows:

(26) $$\begin{array}{l} R_{\alpha }^{2}=\sum_{k=1}^{k_{\max}}{\left\|I_{f_{k}}^{\alpha }A_{f_{k}}Q^{\alpha }-T_{f_{k}}^{\delta }\right\|}_{2}^{2}+\alpha_{TK}Tk\left(Q^{\alpha }\right)+\alpha_{L1}L_{1}\left(Q^{\alpha }\right)+\alpha_{TV}TV\left(Q^{\alpha }\right),\;\\ \;\;\;\;\;\;\;\;\;\;\;\;\;\;\;\;\;\;\;\;\;\;\;\;\;\;\;\;\;\;\;\;\;\;\mathrm{with}\;\;\alpha =\left(\alpha_{TK},\alpha_{L1},\alpha_{TV}\right) \end{array}$$

### 3.2. Inversion Algorithm

The regularization parameters *α_TK_*, *α*_*L*1_, and *α*_*TV*_ in Equation (26) determine how large the different regularization terms are with respect to the discrepancy term. The degree of regularization can be varied by modifying the values of the regularization parameters: large values increase the stability of the inversion process, in the sense that the solution becomes less sensitive to noise in the data, but this stability comes at the expense of introducing an error in the solution.

#### 3.2.1. Regularization Parameters

In order to find the optimum regularization parameters, our choice is to start iterations with rather high initial values, $\alpha_{TK_{0}}$, $\alpha_{L1_{0}}$, and $\alpha_{TV_{0}}$, and reduce them in each iteration according to different decay factors: $\gamma_{TK}=0.3$, $\gamma_{L1}=0.75$, and $\gamma_{TV}=0.75$, respectively. The Tikhonov regularization parameter $\alpha_{TK_{0}}$ decays much faster than $\alpha_{L1_{0}}$ and $\alpha_{TV_{0}}$, so the effect of Tikhonov regularization is basically significant in the first iteration (iteration zero). Tikhonov provides smooth solutions, which is beneficial at the beginning of the inversion and guarantees that the first solution does not get dominated by noise, but sharper solutions are then sought. Moreover, *L*1 and total variation cannot be implemented at the beginning, because they make use of the solution in a previous iteration. Theoretical results [21] suggest that it is prudent to stop minimization iterations before achieving the noise level *δ*. Keeping this in mind, in this problem, we have found that stopping iterations when the minimum discrepancy term is found delivers good results. This is a heuristic stopping criterion, which probably works because we are solving a highly overdetermined problem with quite uncorrelated data noise and gives us optimum results for the retrieved normalized heat source distribution. An important aspect that is worth mentioning about the chosen stopping criterion is that there is no over-fitting of the data, i.e., fitting the noise rather than the underlying function. Regarding the optimum values of the decay factors, there is a lack of theoretical results on this subject. Small values decrease the number of iterations needed to reach the solution, but reduction factors below 0.5 may lead to steps in the discrepancy term being too large for the solution to bet retrieved accurately. The initial values of the regularization parameters as well as their decay factors are chosen by performing systematic batteries of inversions until achieving solutions in a reasonable number of iterations, about 20. Next, we describe the iterative process implemented to find the solution.

#### 3.2.2. Iterations

For the inversion procedure, we use domain decomposition iterations to retrieve the normalized heat source distribution, $Q_{}^{\alpha }$, and the set of intensities, $I_{f_{k}}^{\alpha }$, in successive iterations, known as non-linear Gauss–Seidel iterations by blocks. It is a local minimization method used in bi-linear problems such as this one.

Coming back to our problem,

(27) $$T_{f_{k}}^{\delta }\approx A_{f_{k}}\left[Q_{{}_{f_{k}}}^{\alpha }\right]=I_{f_{k}}^{\alpha }A_{f_{k}}\left[Q^{\alpha }\right]\;\;\;\;\;\;\;\;\;\mathrm{for}\;\;k=1,\;\ldots ,k_{\max}$$

the inversion starts from a zero iteration (different from the rest of the iterations), in which we solve the approximated Equation (27) for each modulation frequency separately, regularized with Tikhonov:

(28) $$Q_{f_{k},0}^{\alpha }={\left(A_{f_{k}}^{*}A_{f_{k}}+\alpha_{TK_{0}}I\right)}^{-1}A_{f_{k}}^{*}T_{f_{k}}^{\delta }$$

where $A_{f_{k}}^{*}$ stands for complex conjugate of $A_{f_{k}}$. Taking into account that the right-hand side of Equation (28) is complex and the heat source distribution being real, we obtain $Q_{f_{k},0}^{\alpha }$ as

(29) Qfk,0α=ReAfk*Afk+αTK0I−1ReAfk*Tfkδ

and calculate a first approximation of the separate intensities as

(30) $$I_{f_{k},0}^{\alpha }=\max\left(Q_{f_{k},0}^{\alpha }\right)$$

The intensities obtained are now introduced as an initial guess in Equation (27), combining all modulation frequencies together,

(31) $$\left[\begin{array}{c} I_{f_{1},0}^{\alpha }\;A_{f_{1}}^{}\\ I_{f_{2},0}^{\alpha }\;A_{f_{2}}^{}\\ \ldots \\ I_{f_{k_{\max}},0}^{\alpha }\;A_{f_{{}_{k_{\max}}}}^{} \end{array}\right]Q_{\left(1\right)}^{\delta ,\alpha }\approx \left[\begin{array}{c} T_{f_{1}}^{\delta }\\ T_{f_{2}}^{\delta }\\ \ldots \\ T_{f_{k_{\max}}}^{\delta } \end{array}\right]$$

regularized only with Tikhonov (using again the initial value of the regularization parameter $\alpha_{TK_{0}}$) to obtain a first approximation of the reconstructed normalized heat source distribution, $Q_{\left(1\right)}^{\delta ,\alpha }$. This finalizes iteration zero. In iteration 1, the Tikhonov regularization parameter is reduced for the first time, whereas the regularization parameters corresponding to L1 and TV, $\alpha_{L1_{0}}$ and $\alpha_{TV}{}_{0}$, are used for the first time with their initial values. $Q_{\left(1\right)}^{\alpha }$ is introduced in Equation (27) for each modulation frequency separately, so that a new set of intensities $I_{f_{k},1}^{\alpha }$ is obtained as

(32) $$I_{f_{k},1}^{\delta ,\alpha }=\frac{\left\|T_{f_{k}}^{\delta }\right\|}{\left\|A_{f_{k}}^{}\left[Q_{\left(1\right)}^{\delta ,\alpha }\right]\right\|}$$

Now, the set of intensities $I_{f_{k},1}^{\alpha }$ is introduced into the equation combining all frequencies together, similarly to Equation (31) but now employing *L*1 and *TV* in addition to Tikhonov, to obtain a second approximation of the reconstructed normalized heat source distribution, $Q_{\left(2\right)}^{\delta ,\alpha }$. The subsequent iterations operate the same way, with successive reductions of the regularization parameters, until the criterion described in Section 3.2.1 is fulfilled.

## 4. Inversions of Synthetic Data

We analyze in this section the performance of the algorithm by inverting synthetic data with added uniform noise, which represents a worse scenario than Gaussian noise. The noise is added to the complex temperature, from which amplitude and phase data are calculated, and is specified as a percentage that represents the ratio of the L2-norm of the noise over the L2-norm of the temperature data. The data are generated for the geometries in Figure 1b,c, using Equations (2) and (3), respectively, with the thermal parameters of AISI 304 stainless steel (*D* = 4 mm^2^/s, *K* = 15 Wm^−1^K^−1^), which is the material our samples are made of. Other geometries are presented as well, in order to illustrate the effect of the regularization functionals. Similarly to the experiments, the data introduced in the algorithm are the amplitude and the phase of the surface temperature at each modulation frequency. As mentioned in Section 2, we combine data obtained at modulation frequencies *f_k_* = 0.05, 0.1, 0.2, 0.4, 0.8, 1.6, 3.2, 6.4, and 12.8 Hz in order to gather precision and penetration data. In the next subsections, we analyze the effect of several factors on the quality of the reconstructions. 

### 4.1. Uniform Heat Flux

**Figure 2:**
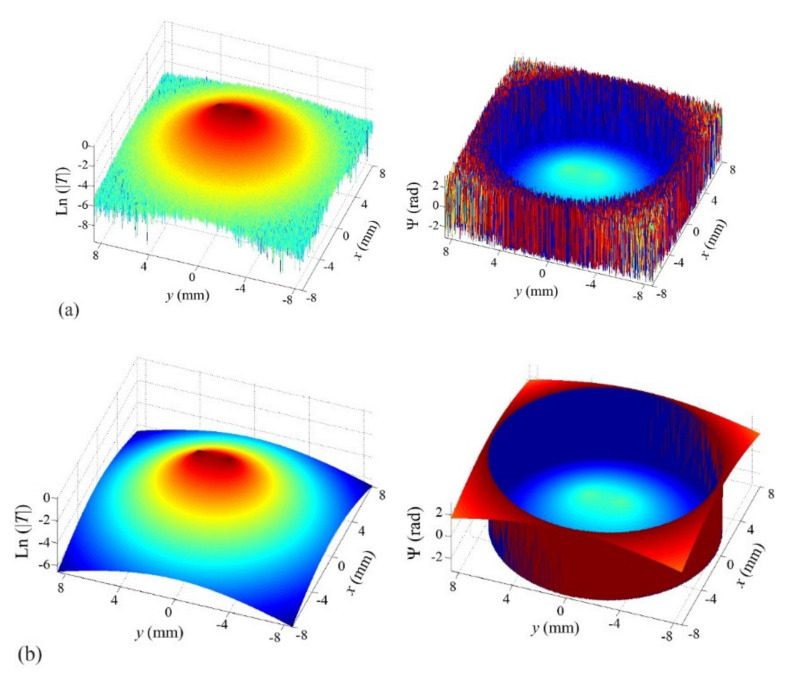
(**a**) Synthetic Ln(|*T*|) (left) and Ψ (right) thermograms with 5% added uniform noise, generated at a modulation frequency of 0.2 Hz, for a homogeneous semicircular open heat source of inner radius *r*_1_ = 1 mm and outer radius *r*_2_ = 1.4 mm buried at a depth *d* = 0.1 mm; (**b**) fitted thermograms.

First, we analyze reconstructions of synthetic data calculated for homogeneous heat fluxes. We start with the geometry of interest, semi-circular bands, as sketched in Figure 1b. In Figure 2a, we show normalized amplitude and phase thermograms calculated for a semi-circular band (*r*_1_ = 1 mm, *r*_2_ = 1.4 mm, *d* = 0.1 mm) at a modulation frequency of 0.2 Hz, with 5% added uniform noise, together with the fitted thermograms (Figure 2b). The discontinuities in the phase are due to the π, −π jump.

**Figure 3:**
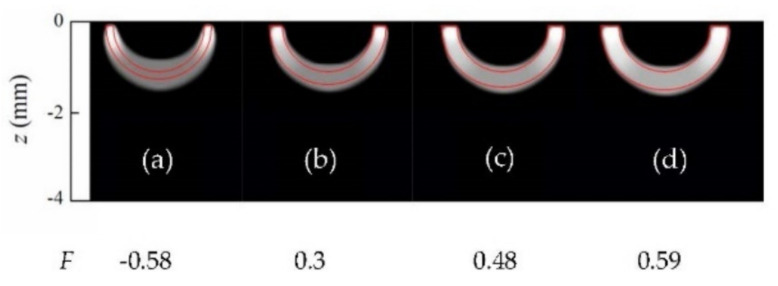
Grey-level representation of the reconstructions of synthetic data with added 5% noise corresponding to homogeneous semi-circular strips of inner radius *r*_1_ = 1 mm and outer radii (**a**) *r_2_* = 1.15; (**b**) *r*_2_ = 1.3; (**c**) *r*_2_ = 1.35; (**d**) *r*_2_ = 1.4 mm. The depth of the upper side is *d* = 0.1 mm in all cases. Real heat source represented by the red contour. Quality factor *F*, under the figure.

The reconstruction is depicted in Figure 3d, together with reconstructions of semi-circular stripes of the same inner radius and smaller outer radii, namely, *r*_2_ = 1.15, 1.3 and 1.35 mm, all with 5% added uniform noise. The reconstructions are displayed in a grey-level representation of the normalized heat flux distribution, white being the maximum value of 1 and black, absence of heat sources. The red line represents the contour of the real heat source that was used to generate the temperature data.

As can be seen, although some “shadowing effect” appears for thin strips, whose tips are brighter than the reconstructed deep central part (fainter and more diffuse), for increasing strip thicknesses, lasso functional works properly and keeps the reconstructed area within the limits of the true heat source, providing very accurate reconstructions. This proves that the methodology is appropriate to identify “hollow” heat sources typically generated by open cracks. 

In order to quantify the quality of the reconstructions, we introduce a quality factor *F*, which takes into account the values of the retrieved $Q_{}^{\alpha }$ inside and outside the real contour:

(33) $$F=\frac{\sum_{i=1}^{M}Q_{i}^{\alpha }-\sum_{j=1}^{P}Q_{j}^{\alpha }}{M}$$

where *M* is the number of nodes within the real geometry and *P* is the number of nodes in plane Π outside the real geometry. According to this definition, *F* = 1 corresponds to a perfect reconstruction. The value of *F* decreases as the quality of the reconstruction worsens, and *F* can reach negative values. If we define an “accurate reconstruction” as one with *F* > 0, we see that the algorithm produces accurate reconstruction for thicknesses above 1.3 mm. Note that F≤0 means that the heat power retrieved from outside the true heat source area is as large as (or larger than) the reconstructed power emitted from inside the true area. 

**Figure 4:**
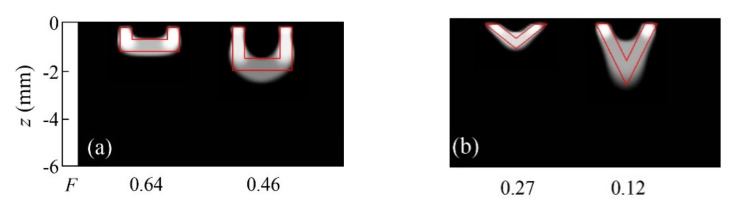
Grey-level representation of the normalized heat source distribution obtained from inversions from synthetic data with 5% added uniform noise corresponding to (**a**) homogeneous rectangular open heat sources; (**b**) triangular open heat sources. In all cases, the upper edges are buried *d* = 0.2 mm. Real contours depicted in red, and values of the quality factor *F* under each reconstruction.

In order to further illustrate the effect of the regularization functionals, we also inverted synthetic data generated for “hollow” heat sources (as in Figure 3), but whose contours feature sharper corners, rather than being smooth circular lines. Figure 4 displays reconstructions of synthetic data with 5% added noise corresponding to uniform heat sources, with the shapes indicated by the red lines.

The first notable feature of Figure 4 is that all reconstructions feature rounded contours. This illustrates the impact of the presence of *TV* regularization in the inversion. As mentioned in Section 3.1, *TV* penalizes the norm of the derivative of the solution, which yields rounded shapes, as the circle is the figure with the shortest contour for a given area. Despite this bias in the reconstruction, and some shadowing effect for the deepest cases (Figure 4a right and 4b right), the hollow region in the middle is identified, and the quality factors are all above the cutoff value.

**Figure 5:**
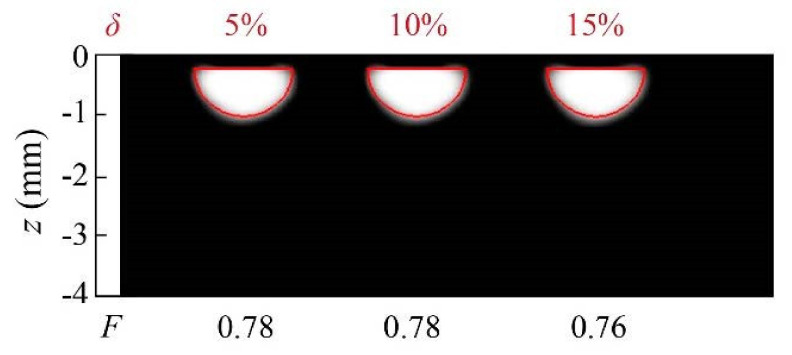
Grey-level representation of the normalized heat source distribution in inversions from synthetic data affected by 5%, 10% and 15% uniform noise, corresponding to a semi-circle of radius *r*_2_ = 0.8 mm, buried at *d* = 0.2 mm. Real contours depicted in red, values of the noise level in the data and quality factor *F* on top of and under each reconstruction, respectively.

To finish the analysis of homogeneous heat sources, we consider the case of kissing half-penny cracks, as a particular case of the geometry depicted in Figure 1b, with *r*_1_ = 0. In Figure 5, we present reconstructions of synthetic data calculated for a kissing half-penny crack (*r*_1_ = 0, *r*_2_ = 0.8 mm, *d* = 0.2 mm) with different noise levels: 5%, 10% and 15%. As can be observed, the inversion algorithm is robust to noise, as the noise level does not significantly affect the quality of the reconstructions. It is worth mentioning that the adequate confinement of the heat sources in all the reconstructions presented is due to the effect of L1 regularization. 

### 4.2. Inhomogeneous Heat Flux

**Figure 6:**
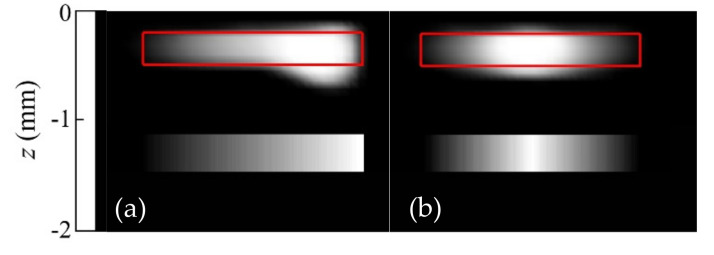
Grey-level representation of the normalized heat source distribution in inversions from synthetic data affected by 5% uniform noise corresponding to an inhomogeneous rectangular heat source *w* = 2 mm and *h* = 0.3 mm, buried at a depth *d* = 0.2 mm, with variable flux in horizontal direction. (**a**) Monotonic linear variation; (**b**) Symmetric linear variation from the center to the edges. Real contours depicted in red.

Let us analyze now how the algorithm performs with inhomogeneous heat fluxes. We start with a simple geometry, the rectangles depicted in Figure 1c. In Figure 6, we present reconstructions of heat sources with the shape of wide and short rectangles (*w* = 2 mm, *h* = 0.3 mm, *d* = 0.2 mm) with linear variations of the flux in the direction parallel to the surface: a monotonic linear variation along the width and a linear variation from the center to the edges. The real heat sources used to generate the data are displayed under each reconstruction.

The results show that the inversion algorithm is able to retrieve smooth flux variations parallel to the surface qualitatively. This result is surprising, as *TV* favours blocky reconstructions. We believe that the “soft” approximation that we use to introduce *TV* (Equation (25)) from the first-order Tikhonov functional helps in identifying these flux gradients. 

**Figure 7:**
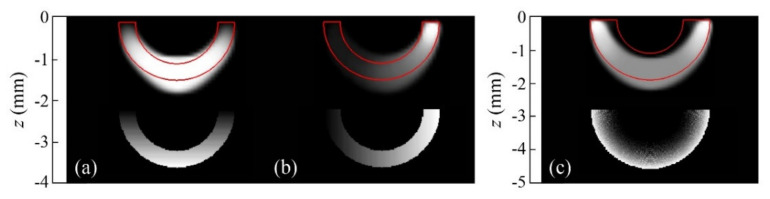
Grey-level representation of the normalized heat source distribution inverting synthetic data affected by 5% uniform noise, corresponding to semi-circular open heat sources, buried at a depth *d* = 0.1 mm. Three linear inhomogeneous fluxes are considered: (**a**) flux varying with depth (*r*_1_ = 1 mm and *r*_2_ = 1.4 mm); (**b**) flux varying with angle (*r*_1_ = 1 mm and *r*_2_ = 1.4 mm); (**c**) flux varying with radius (*r*_1_ = 1 mm and *r*_2_ = 1.8 mm). Real contours depicted in red, and real heat source distributions represented under each reconstruction.

Finally, we consider flux variations in the geometry of interest, the semi-circular stripes of Figure 1b, representing open half-penny cracks. Figure 7 displays reconstructions of synthetic data with 5% noise corresponding to stripes of inner and outer radii *r*_1_ = 1 mm and *r*_2_ = 1.4 mm, respectively, with linear depth and angular variations of the flux. We also show the inversion corresponding to a strip of the same inner radius, *r*_1_ = 1 mm, but a larger outer radius, *r*_2_ = 0.8 mm, featuring a linear radial dependence of the flux. In all cases, the depth is *d* = 0.1 mm.

As for flux varying with depth, it is clear that the absence of heat emitted from the shallower tips allows the central deep part to dominate the reconstruction, and thus, the shadowing effect disappears. In any case, the flux dependence is identified in the reconstruction. 

Regarding flux varying with angle, the algorithm behaves very well: the right tip is accurately defined, and the variation from maximum to null flux is nicely recovered, just as in the case of the geometry shown in Figure 6a, which further proves that flux variations with a main component parallel to the surface can be accurately retrieved in wide and narrow geometries. Lastly, the radial dependence of the flux is not identified. 

From the previous analysis, we can conclude that short-distance variations (such as the radial dependence in Figure 7c) cannot be identified by the algorithm. However, long-distance variations of the flux in narrow figures are qualitatively identified, especially if the variation is parallel to the sample surface. 

## 5. Experiments and Inversions of Experimental Data

**Figure 8:**
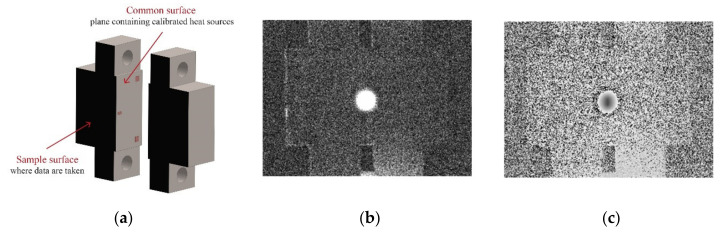
(**a**) Diagram of the AISI 304 stainless steel sample open with a calibrated heat source on the common surface between the two twin parts; (**b**) amplitude and (**c**) phase thermograms obtained at 6.4 Hz with a semi-circular Cu film of radius *r*_2_ = 0.8 mm, buried at a depth of *d* = 0.1 mm.

We have checked the ability of the inversion algorithm to reconstruct open and non-uniform heat sources by inverting experimental data obtained on samples that generate calibrated heat sources under ultrasonic excitation. The samples were described elsewhere [20]. They basically consist of two twin AISI 304 stainless steel parts (*D* = 4 mm^2^/s, *K* = 15 Wm^−1^K^−1^), each machined with one flat and polished surface (the “common surface” in Figure 8a). A Cu foil, 38 μm thick, of calibrated dimensions is sandwiched between the common surfaces at a well-known distance (depth) from the surface where data are taken. The two steel parts are joined together by means of screws. Under the action of ultrasounds, the friction of the Cu foil with the steel surfaces produces a calibrated heat source. With the aim of generating a controlled homogeneous flux, two more Cu slabs of the same thickness are introduced at the deeper far ends of the surface containing the calibrated heat source (far enough from the surface where data are taken so as not to disturb the temperature field at the front surface). This guarantees a reasonable parallelism of the common surfaces and homogeneity of the induced heat sources. A sketch of the samples is shown in Figure 8a, together with examples of amplitude and phase images obtained at a modulation frequency of 6.4 Hz for a rectangular Cu slab of width *w* = 1.4 mm and height *h* = 2.3 mm, buried at a depth of *d* = 95 μm.

We excite the sample by means of UTVis equipment from Edevis, tuneable between 15 and 25 kHz, with maximum power of 2.2 kW at 20 kHz. We work at 23 kHz, the optimum frequency for our samples, and we modulate the ultrasound’s amplitude at much lower frequencies, namely, 0.05, 0.1, 0.2, 0.4, 0.8, 1.6, 3.2, 6.4, and 12.8 Hz, by means of a function generator. The modulation is carried out by means of a function generator, whose output is fed into the ultrasounds generator. A reference signal coming from the function generator serves as a reference for the lock-in analysis. The sample is supported by a Teflon block, and a thin tape of Al is inserted between sample and the sonotrode to improve mechanical coupling.

**Figure 9:**
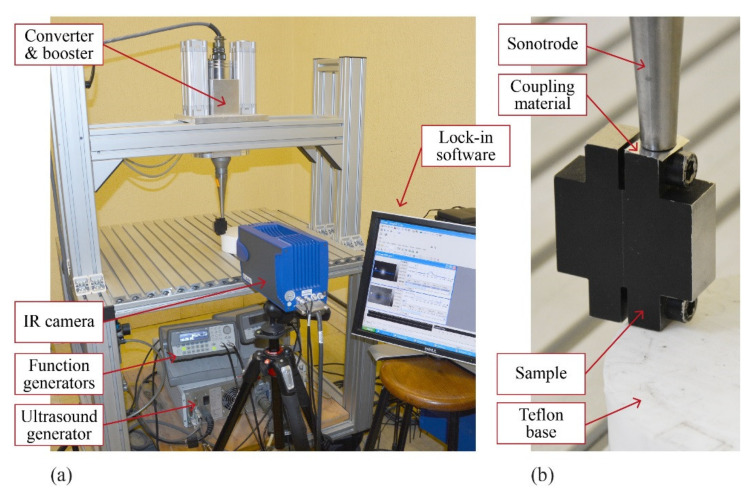
(**a**) Picture of the excitation system; (**b**) detail of the sample closed and in contact with the sonotrode for excitation.

The surface of the material is covered with a thin layer of high-emissivity paint, and the infrared radiation emitted by the surface is collected by an IR video camera (JADE J550M, from Cedip, France) equipped with a 320 × 256 pixel InSb detector working in the 3.5–5 μm range with NETD of 25 mK. The camera lens has a focal length of 50 mm. Placing the sample at the minimum working distance, we achieve a spatial resolution of 135 μm. A picture of the experimental set-up can be seen in Figure 9. 

Modulating the ultrasound’s amplitude and applying lock-in processing to the image sequence reduces the noise in phase and amplitude images and allows applying very limited ultrasound power (between 25 and 50 W, depending on the modulation frequency). We typically analyse sequences of 20000 images at a frame rate of 320 images per second (at half frame), which reduces the average noise in amplitude images down to 0.4 mK in about 1 min acquisition time, well below the noise equivalent temperature difference (*NETD*) of the camera, according to [22]:

(34) $$\langle A_{amp}\rangle =2\frac{NETD}{\sqrt{N_{images}}}$$

This is beneficial to reaching the steady state quickly and to preventing any damage to the sample. In experiments with real samples, this noise reduction also enables detecting deep or small heat sources that produce week signals.

**Figure 10:**
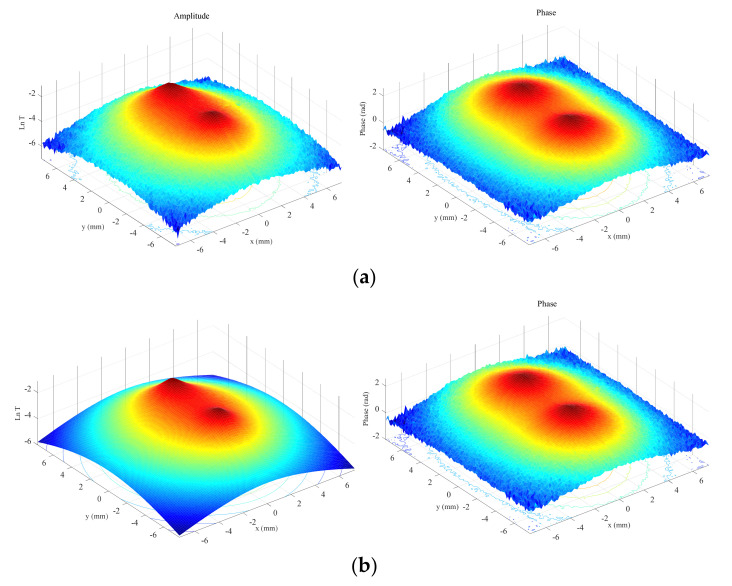
(**a**) Experimental natural logarithm of amplitude (left) and phase (right) obtained for a sample containing a semi-circular Cu strip of inner radius *r*_1_ = 1.2 mm and outer radius *r*_2_ = 2 mm buried at *d* = 0.32 mm, obtained at 0.2 Hz; (**b**) fitted thermograms.

As an example of experimental data, in Figure 10, we show experimental amplitude and phase thermograms corresponding to a semi-circular Cu strip of inner radius *r*_1_ = 1.2 mm and outer radius *r*_2_ = 2 mm buried at a depth of *d* = 0.32 mm, obtained at 0.2 Hz.

**Figure 11:**
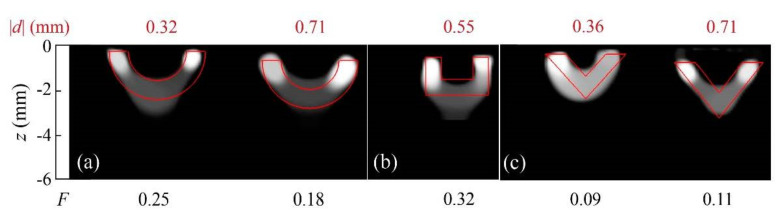
Grey-level representation of the normalized heat source distribution in inversions from experimental data corresponding to (**a**) semicircular Cu bands of inner and outer radii *r*_1_ = 1.2 mm and *r*_2_ = 2 mm, respectively, buried at depths *d* = 0.32 and 0.71 mm; (**b**) a square Cu band of outer width 2.8 mm, outer height 1.7 mm, and thickness 0.7 mm buried at a depth *d* = 0.55 mm; (**c**) triangular Cu bands of outer width 3.6 mm, outer height 2 mm, and thickness 0.9 mm buried at depths *d* = 0.36 and 0.71 mm. Real contours depicted in red, and values of the depth of the heat sources and quality factor *F* on top and under of each reconstruction, respectively.

The reconstruction obtained by combining data taken in the whole frequency set (0.05 up to 12.8 Hz) is depicted in Figure 11, together with a reconstruction of the same slab buried at *d* = 0.71 mm and reconstructions corresponding to other open heat source geometries.

The results confirm some of the features observed in the inversion of synthetic data. On the one hand, rounded contours dominate the reconstructions, which, as explained in Section 4.1, is due to the presence of a *TV* term in the regularization penalty. Furthermore, the shadowing effect is visible, due to the stronger contribution of the shallowest heat sources that dominate the reconstruction. Nevertheless, in all geometries, the deeper central areas correctly show the path the bands follow, and all depths are well-recovered. The quality factors are in all cases above the cutoff value of *F* = 0.

**Figure 12:**
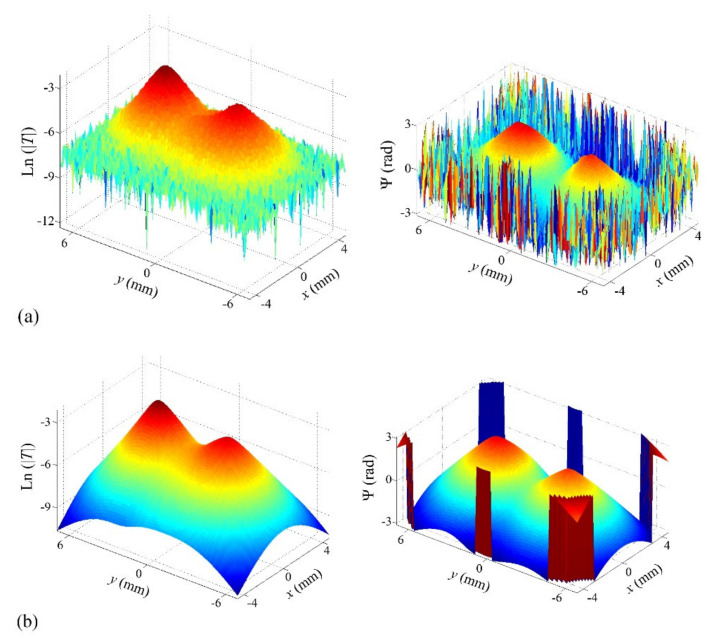
(**a**) Experimental natural logarithm of amplitude (left) and phase (right) obtained at a modulation frequency of 1.6 Hz in a sample containing two quarters of circular strips made of stainless steel and W (both 25 μm thick) with inner and outer radii *r*_1_ = 4.2 mm and *r*_2_ = 5.1 mm, respectively, buried at a depth of *d* = 0.16 mm below the surface; (**b**) fitted thermograms.

Next, we tried to obtain experimental data corresponding to inhomogeneous heat sources. As mentioned above, our samples are intended to produce homogeneous heat sources, so we decided to take data combining in the same experiment two strips with the shape of a quarter of a circle to form a semi-circular band with two homogeneous but different heat fluxes on its two halves. In Figure 12, we present the experimental amplitude and phase thermograms obtained by combining two quarters of circular strips made of stainless steel and W (both 25 μm thick) with inner and outer radii *r*_1_ = 4.2 mm and *r*_2_ = 5.1 mm, respectively, buried at a depth of *d* = 0.16 mm below the surface, at a modulation frequency of 1.6 Hz. Unfortunately, we do not have an independent estimate of the ratios of fluxes generated by the two halves in these combinations.

**Figure 13:**
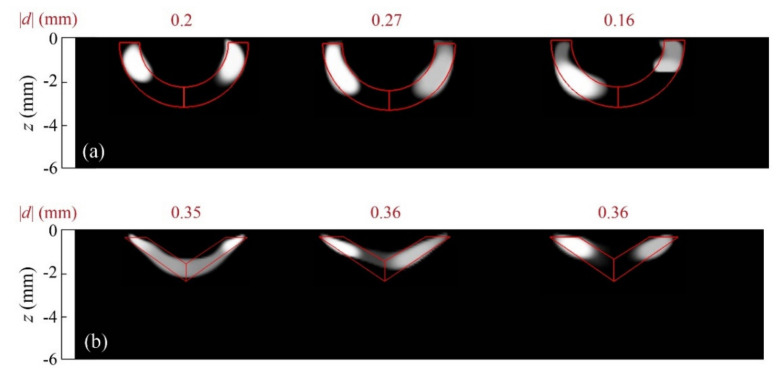
Grey-level representation of the normalized heat source distribution of inversions from experimental data corresponding to (**a**) two quarters of circular bands of inner radius *r*_1_ = 4.2 mm and outer radius *r*_2_ = 5.1 mm buried at depths *d* = 0.2, 0.27, and 0.16 mm and (**b**) two halves of triangular bands of outer width 5.6 mm, outer height 2.4 mm, and thickness 0.9 mm, buried at depths 0.35 and 0.36 mm. For both geometries, the material combinations for the left and right halves of the bands are the following: 38 µm thick annealed Cu and hard Cu foils (left), 25 µm thick Cu and stainless steel foils (centre), and 25 µm thick stainless steel and W foils (right). Real contours depicted in red and values of the depth of the heat sources on top of each reconstruction.

The reconstruction obtained by combining amplitude and phase data in the whole frequency set is depicted in Figure 13a (right), together with two more reconstructions, from data obtained using other combinations of materials: on the left, annealed and hard Cu foils, both 38 μm thick, and at the center, 25 μm thick hard Cu and stainless steel foils. In Figure 13b, we display the reconstructions obtained for the same material combinations but with triangular geometries.

As may be noted, for either geometry, similar results are obtained regarding the heat flux generated by each material combination: the annealed and hard Cu halves (left) act as a homogeneous heat source, whereas differences in the retrieved heat source distribution are more significant for the other two material combinations: Cu–stainless steel (center) and stainless steel–W (right). These differences in the retrieved fluxes are similar for both geometries, which proves the consistency of the inversions. Although the shadowing effect makes the retrieved areas miss the contribution of the central deeper positions in the deepest cases, the overall geometry and the depths of all heat sources are well-recovered. These results prove that differences in the heat flux distributions can be qualitatively characterized with the proposed algorithm.

## 6. Summary and Conclusions

In this work, we have demonstrated that multi-frequency lock-in vibrothermography data in combination with a least-squares minimization algorithm regularized by *TV* and lasso functionals allows characterizing ”hollow” non-compact vertical heat sources typically generated by real open cracks in vibrothermography experiments. We have obtained semi-analytical expressions of the surface temperature distribution generated by vertical heat sources with the shape of semi-circular stripes, representing the behavior of open half-penny cracks excited with ultrasounds. A detailed description of the regularization strategies (starting from truncated SVD to Tikhonov, total variation, and lasso) as well as of the inversion algorithm has been presented, and we have proposed a criterion to evaluate the quality of the reconstructions. The inversions of synthetic data with added noise show that the algorithm is able to identify “hollow” uniform heat fluxes and reveal that when the heat source spans a large range of depths, the reconstructions are affected by the shadowing effect, which blurs the deepest part of the heat source, due to the stronger contribution of shallow locations. Inhomogeneities in the heat flux are qualitatively identified except in the case of radial dependence of the flux. The predictions of the reconstructions with synthetic data were confirmed by inversions of experimental data taken on calibrated samples. The results confirm that it is possible to characterize the shape of heat sources generated by open cracks is lock-in vibrothermography experiments. The lock-in processing of modulated data allows detecting signals below the NETD of the camera. The possibility of identifying the regions of the crack that produce heat and the distribution of these heat sources in lock-in vibrothermography open the way to understanding the configuration and dynamics of cracks in this kind of experiment.

## Data Availability

The data are available under reasonable request to the corresponding author.
